# Interactions with Combined Chemical Cues Inform Harvester Ant Foragers' Decisions to Leave the Nest in Search of Food

**DOI:** 10.1371/journal.pone.0052219

**Published:** 2013-01-08

**Authors:** Michael J. Greene, Noa Pinter-Wollman, Deborah M. Gordon

**Affiliations:** 1 Department of Integrative Biology, University of Colorado, Denver, Colorado, United States of America; 2 Department of Biological Sciences, Stanford University, Stanford, California, United States of America; University of Western Ontario, Canada

## Abstract

Social insect colonies operate without central control or any global assessment of what needs to be done by workers. Colony organization arises from the responses of individuals to local cues. Red harvester ants (*Pogonomyrmex barbatus*) regulate foraging using interactions between returning and outgoing foragers. The rate at which foragers return with seeds, a measure of food availability, sets the rate at which outgoing foragers leave the nest on foraging trips. We used mimics to test whether outgoing foragers inside the nest respond to the odor of food, oleic acid, the odor of the forager itself, cuticular hydrocarbons, or a combination of both with increased foraging activity. We compared foraging activity, the rate at which foragers passed a line on a trail, before and after the addition of mimics. The combination of both odors, those of food and of foragers, is required to stimulate foraging. The addition of blank mimics, mimics coated with food odor alone, or mimics coated with forager odor alone did not increase foraging activity. We compared the rates at which foragers inside the nest interacted with other ants, blank mimics, and mimics coated with a combination of food and forager odor. Foragers inside the nest interacted more with mimics coated with combined forager/seed odors than with blank mimics, and these interactions had the same effect as those with other foragers. Outgoing foragers inside the nest entrance are stimulated to leave the nest in search of food by interacting with foragers returning with seeds. By using the combined odors of forager cuticular hydrocarbons and of seeds, the colony captures precise information, on the timescale of seconds, about the current availability of food.

## Introduction

Social insect colonies operate without central control. Individual colony members change behavior in response to local cues. These responses, in the aggregate, allow the colony to adjust to changing conditions and colony needs [1]. For example, in many social insect species, foragers are stimulated to leave the nest in search of food by interactions with other workers. In *Polybia* wasps, foragers are stimulated to leave the nest in response to ‘biting’ [2]. In stingless bees (*Melipona*), foragers leave the nest in response to returning foragers carrying food [3]. In honey bees, foragers are stimulated by a variety of interactions including antennal contact [4], interactions with bees inside the nest that unload food from returning foragers [5], and the odor of flowers recently visited [6]. To regulate foraging in response to food availability, the cues that influence foraging activity must correspond to external conditions.

In the seed-eating red harvester ant *Pogonomyrmex barbatus*, no ant makes any global assessment of food availability, but the rate at which successful foragers return to the nest entrance reflects the time it takes to find food [7]. Each forager leaves the nest in a stream of foragers, travels quickly for up to 20 m from the nest, then searches individually for a seed, and returns directly to the nest as soon as it finds food [8]. The duration of its trip depends mainly on search time, not on the distance travelled [9]. The more food is available, the less time is needed to search and the more quickly a forager returns with food. Thus the overall rate of return of successful foragers reflects the availability of food on that day. Unlike many other ant species, *P. barbatus* foragers are not normally recruited to patchy food sources in the field using chemical recruitment trails [10]. Seeds are scattered in the soil and retrieved individually [11]. The direction of foraging is influenced by chemicals from the Dufour's gland placed on the nest mound by another task group, patrollers, and by a forager's memory of where it last collected a seed [9]; [12].

Here we examine how *P. barbatus* foragers, returning to the nest entrance with food, stimulate outgoing foragers to leave the nest to search for food. Previous work suggested that both the arrival of foragers and the arrival of food are crucial to stimulate foraging activity. When returning foragers were deprived of their food and allowed to return to the nest, foraging activity slowed [13]. When foragers with food were prevented from returning to the nest, foraging activity slowed, but when foragers without food were prevented from returning, there was no effect on foraging activity [7]. Observations of *P. barbatus* colonies with a videoscope show that when the returning foragers enter the nest, they go into a short entrance tunnel that leads down to an entrance chamber (Gordon, unpublished data). The entrance chamber serves as an area of high interaction among workers with the entrance to the tunnel leading into the nest from the chamber serving as an interaction hotspot [14]. Somewhere along the way to the entrance chamber, each returning forager drops its seed, and then becomes an inactive, outgoing forager waiting to leave on its next trip. The seeds are taken further down into the nest by other ants. In combination, this previous work suggests that the rate at which an inactive, outgoing forager interacts with returning foragers carrying food determines how soon any awaiting forager goes out again on its next trip.

Mimics can be used to substitute ants to stimulate foraging activity in *P. barbatus* colonies [15]–[16]. Here, using mimics, we asked whether interactions in the nest entrance with the odor of foragers, odor of seeds, or a combination of both, can modulate the activation of foragers. We hypothesized that the addition to nest entrances of the mimics coated with a combination of seed odor and forager odor would increase foraging activity, but that seed odors alone, corresponding to food alone, and forager odors alone, corresponding to foragers without food, would not increase foraging activity. We examined whether interaction rate increases in response to a combination of forager and seed odors, relative to the rate of interaction with blank controls.

## Materials and Methods

To mimic the return of foragers, seeds, or both to the nest, we used forceps to manually introduce mimics, treated small alumina chips (8–14 mesh; Fisher Scientific), into the entrance tunnel just inside the nest entrance during the peak in foraging activity. A returning forager interacts with many other ants, including inactive foragers, in the entrance tunnel and a deeper chamber. The mimics were approximately the size of some of the seeds that the ants collect [11], easily carried by the ants, and were chemically inert to organic solvents.

All colonies were treated with mimics with the following odors: 1) blank, 2) forager odor (cuticular hydrocarbons), 3) seed odor (oleic acid), and 4) the combination of forager and seed odors. We compared the colony's foraging activity before and after the addition of the mimics for each mimic type.

Blank control mimics were created for each colony by soaking 300 alumina chips in 2 ml of 100% pentane. The solvent was allowed to evaporate. This mimic controlled for any effects that residual solvent odors, if any, may have had on ant behavior, and allowed us to determine if the addition of mimics alone mechanically stimulates increased levels of foraging.

To create forager mimics, forager cuticular hydrocarbons were extracted and isolated from frozen foragers. Long-chain hydrocarbons are unreactive and not susceptible to evaporation in storage; freezing does not significantly cause quantitative or qualitative changes in cuticular hydrocarbon profiles compared to fresh samples [17]. To avoid responses to non-nestmates, forager mimics used at a given colony were made using extracts from ants of the same colony. Cuticular hydrocarbons contain cues about task identity in *P. barbatus* [15]; [18], so hydrocarbons were extracted only from foragers. To extract cuticular hydrocarbons, 20 frozen foragers from each colony were thawed and soaked in 1 ml of 100% pentane for 10 minutes [15]–[16]; [19]. Cuticular hydrocarbons were isolated from polar surface lipids by running the surface lipid extracts through a silica gel column using 100% pentane as the eluent [15]–[16]. The 20 ant-equivalents of cuticular hydrocarbons in pentane were added to 300 alumina chips and the solvent was allowed to evaporate, thus coating the chips with forager cuticular hydrocarbons. An ant-equivalent of cuticular hydrocarbon for *P. barbatus* is 9 ng of hydrocarbon per ant [20]. Given that a mimic has a much smaller surface area compared to a live ant, we estimated that 20 ant-equivalents per 300 mimics would coat each mimic with a biologically relevant amount of hydrocarbon.

Seed mimics were created by soaking 300 chips in 2 ml of a 20% oleic acid (volume:volume; Fisher Scientific) solution dissolved in 100% pentane. The solvent was subsequently allowed to evaporate from the chips. Oleic acid was chosen as a seed odor because it is treated as a food odor by red harvester ants during periods of high foraging activity [21]. The amount of oleic acid added to each mimic was chosen to estimate the amounts found per gram of seeds [22].

Mimics coated with both forager and seed odors were produced by soaking 300 alumina chips in 20 foragers' worth of cuticular hydrocarbons and 2 ml of the 20% oleic acid solution in pentane. The solvent was allowed to evaporate from the chips before use in the experiment. This design ensured that the same amount of cuticular hydrocarbon was applied here to mimics as on the forager odor mimics and oleic acid was applied in similar amounts as on the seed odor mimics.

Three forager mimic samples, three seed mimic samples, and three samples of mimics coated with both forager and seed odor were later analyzed in the laboratory using gas chromatography to confirm amounts of cuticular hydrocarbon and/or oleic acid added to mimics. Mimics were extracted for 20 minutes in 1.0 ml of 100% pentane. To each extract, 40 micrograms of n-dotriacontane standard (ULTRA Scientific) was added. To samples containing oleic acid, 10 microlitres of Bis(trimethylsyiyl) trifluoroacetamide (BFTA; Restek) was added and allowed to react for 20 minutes. Pentane was allowed to evaporate from all samples and 8 microliters of 100% pentane was added to each sample. Eight microliters of sample were then injected into the gas chromatograph. Analysis was conducted using a Varian 3900 gas chromatograph with a DB-5 fused silica capillary column (30 m, 0.25 um ID, 0.25 um film thickness; J&W Scientific). Oven temperature was held at 170°C for 5 min during injection, raised to 220°C at 25°C per min, and then to 310°C at a rate of 3°C·min per min with a 5 min hold. Peak areas were measured by integration of peaks. Oleic acid was identified by comparison to the elution time of an oleic acid standard. Hydrocarbon peaks were identified by comparison to n-alkane standards and known elution patterns and retention times. Oleic acid and hydrocarbons were quantified by comparing to the peak area of the n-dotriacontane standard. One sample of the combination of forager and oleic acid treatment was not included in the analysis because of problems with chromatography.

We conducted the experiment during August 2008 and August 2010 on a total of 23 colonies. The experiment was conducted at a long-term study site near Rodeo, NM, with a population of colonies of known age [8]. We chose focal colonies that habitually had only one or two trunk foraging trails. *Pogonomyrmex barbatus* workers leave the nest entrance along trunk trails that extend several meters from the nest, from which the foragers spread out to collect seeds by searching or digging in the soil.

No specific permits were required for the described field studies. Permission for use of the site was granted by Stanford University.

In 2008, replicates were conducted over 4 days, from August 20 to August 24 using 15 mature colonies ranging in age from 5 to 24 years. On each day, a colony received the four types of mimics sequentially in a random order with a three-minute break between trials. We performed experiments on 3 to 5 colonies per day, always during the time of peak foraging activity, when returning and outgoing forager rates were about equal.

In 2010, replicates were conducted from August 20 to August 24 using 8 colonies ages 4 to 14 years old, all different colonies from those used in 2008.

To control for day-to-day variation in foraging activity (e.g. [23]), all colonies received the same stimulus each day. Blank controls were offered on the first day of the experiment to provide the most conservative interpretation of the results, as the data from 2008 showed that when different treatments were provided on the same day, there was a non-significant trend for the first addition of mimics, whatever the treatment, to elicit an increase in foraging. For 2010, the order of presentation for the other mimics was chosen by selecting at random a tube from a rack of tubes. All colonies tested on that day were presented mimics of the type in the randomly selected tube. The order of treatments was: mimics treated with both forager cuticular hydrocarbons and oleic acid were introduced on the second day, forager cuticular hydrocarbons on the third, and oleic acid on the fourth day. There were no obvious differences in daily conditions, for example for rainfall, over the experiment in 2010 and, although such a design can potentially introduce bias to the experiment, we found no differences between years in responses by colonies to each treatment.

In each trial, one observer recorded the number of foragers moving away from the nest along a foraging trail across an invisible line. We chose to measure foraging activity as the rate of outgoing foragers because previous work has shown that the majority of foragers return with seeds to the nest [11]. The line, marked by flags, was about 1 meter from the nest entrance. For colonies with more than one foraging trail, observations were made at the trail with the highest foraging rate in 2008 and at both trails in 2010. A second person added mimics to the nest entrance. All trials were conducted at about the same time each morning, about 30 minutes between start times, when the colony had reached peak foraging activity and returning and outgoing forager rates were about equal.

In 2008, recordings of numbers foraging were made by pressing a button on a cell phone programmed to record the time of data entry, each time 5 foragers passed the marked line. In 2010, the number of foragers travelling away from the nest on a foraging trail across an invisible line was recorded by video at a site about 0.5 m from the outer edge of the nest mound, or about 1 m from the nest entrance. If foragers were travelling from the nest along two trails, foraging rates were recorded on both and numbers of foragers were combined. We used AnTracks image analysis software, developed by Martin Stumpe (http://antracks.martinstumpe.com), to track all individual ants in the video and used the tracks to count foraging rates.

**Figure 1:**
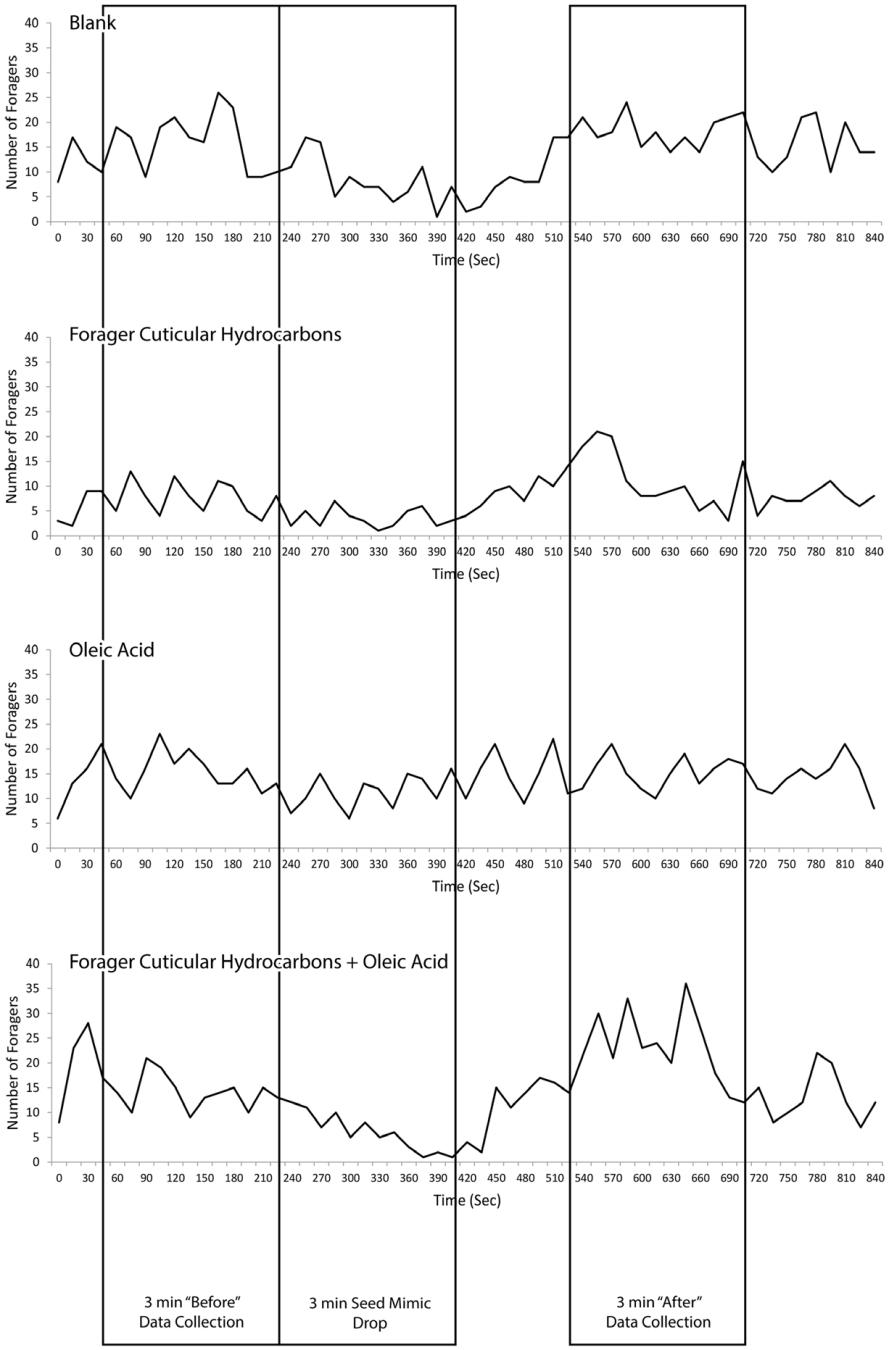
Number of foragers leaving the nest along foraging trails for one representative colony. Also shown are time periods during which data were collected and seed mimics were added to colonies.

The number of foragers moving away from the nest on the trail was recorded for 3 min before the addition of mimics. In 2008, mimics were then added to the nest entrance at a rate of about 1 per 2 sec, for 1 min and in 2010, 100 mimics were added over a 3 minute duration, a rate of about 1 per 1.8 seconds. These rates were near the median rate at which foragers returned in other observations [24]; the highest rate at which foragers were observed to enter the nest was about 5 per sec. It appeared that mimics were not large enough to stop the flow of returning foragers entering the nest, although there was consistently a short-lived decline, lasting 1–2 min, in the flow of outgoing foragers leaving the nest immediately after mimics were dropped into the entrance (Figure 1). Figure 1 shows the response to all treatments of one representative colony in 2010, and shows the temporary decline in foraging due to the addition of the chips. Ants coming to the entrance from inside the nest immediately picked up the mimics in their mandibles and took them deeper into the nest. After mimics were dropped into the nest, the number of outgoing foragers moving away from the nest was recorded.

To analyze the data, we compared the total number of foragers counted during a 3 min interval before mimics were added to the total number of foragers during a 3 min interval after mimics were added using a paired t-test. Because we conducted four tests, one for each treatment, we used a Bonferroni correction for multiple testing to set significance level at p value = 0.0125. Data after mimics were added were collected beginning 2 min after the last mimic was added, because previous work showed a delay of about 1–2 min in a change in foraging activity in response to a change in the rate of forager return [23]–[24] and because of a temporary decline in foraging activity immediately after the addition of chips (see, Figure 1). We found no differences between years in each treatment, comparing 2008 and 2010 using a Mann-Whitney test: there was no difference in response to the addition of blank mimics (p = 0.428), oleic acid (p = 0.09), forager CHC (p = 0.115), or combined oleic acid and forager CHC (p = 0.591). Therefore, in further statistical analyses, we combined data for the two years to improve statistical power. We first used paired t-tests to compare the numbers of foragers before and after addition of mimics. We then used repeated-measures ANOVA to compare the change in numbers of foragers counted during observation periods before and after addition of mimics among the 4 treatments.

To examine how foragers inside the nest entrance interacted with mimics, we observed the response of foragers inside the nest to mimics coated with both forager odor and oleic acid, and to blank mimics. Mimics were made as above. Observations were made with two colonies in August 2011. To examine interactions inside the nest, we created a transparent ceiling above the nest entrance. The soil above the nest entrance area was scraped away, using a spoon, to expose the top part of the nest entrance and entrance tunnel. An opaque 20×25×2.5 cm wood block was placed on top of the excavated region, creating a ceiling. The colonies were left undisturbed for 2–3 days prior to running the experiments. On the morning of each experiment, at least 30 minutes before starting an experiment, we replaced the wood block ceiling with a transparent glass sheet (20×25×0.2 cm) which was kept covered with the wood block until the experiments began. Ants did not appear to be disturbed when the wooden cover was replaced by the glass. During the experiments, the nest mound was shaded using a beach umbrella and the wood block cover was removed from the glass. The glass sheets were rinsed in a dilute detergent solution the night before the experiment to prevent condensation of water during data collection. At the end of each day's experiments, the transparent glass ceiling was replaced with the opaque artificial ceiling.

A video camera, set up above the glass sheet, was used to record interactions between workers and mimics in the region of the nest entrance exposed to view. Ants in the exposed area could either leave the nest through the nest exit or go further down a tunnel leading deeper into the nest. While colonies were foraging, we filmed the area for 2 minutes. We then added into the nest entrance 15 mimics during a 30 second period, at a rate of 1 mimic per 2 seconds. In each trial, we first added blank mimics and then after an interval of at least one hour, we added mimics coated with the combination of forager odor and oleic acid, and then recorded for another 3.5–5.5 min. After 6–8 min, mimics were removed from the nest entrance. Each colony was tested once and both colonies were tested on the same day.

To measure interaction rate with the blank and combined forager/oleic acid mimics, we selected from the video 10 foragers from the 2 min before mimics were added and another 10 foragers from 2 min of observation after mimics were added beginning 30 seconds after the last mimic was added. The foragers selected before and after mimics were added are probably different individuals, because forager turnover occurs on a longer timescale than the span of 5 minutes over which the two samples of forager were chosen. The number of foragers in a colony is large [25], and the mean duration of a foraging trip is about 20 min [8]. An interaction was recorded when the focal individual's head came within the length of an ant's antenna or less from another ant or from a mimic. Each forager's interactions with other ants or with mimics were recorded using a Matlab script (code available upon request) that records the frame and position of user-identified events. Each of the 20 foragers was tracked from the time it appeared in the nest entrance, under the glass sheet, until it left the nest (ranged in duration from 1–72 seconds, mean±SD: 11.35±10.96). Foragers were selected for analysis only if their entire trajectory could be observed without obstruction. We then calculated an interaction rate for each forager as the number of interactions divided by its duration in the nest entrance. We compared forager interaction rates with blank mimics, combined forager/oleic mimics, and ants, using four ANOVAs, one for each of the following dependent variables: interaction rate with ants only during the 1. blank or 2. combined forager/oleic mimics treatments; and interaction rate with both ants and mimics during the 3. blank or 4. combined forager/oleic mimics treatments. In all 4 ANOVAs the independent variables were time period (before or after mimics were added), and colony, and were treated as categorical fixed effects. We then evaluated the effect of treatment, blank versus combined forager/seed odor on interactions only with mimics, using an ANOVA, with interaction rate with mimics as the dependent variable and treatment and colony as the independent variables, treated as categorical fixed effects. The hypothesis we tested was that interactions with the combined forager/oleic mimics treatments will be equivalent to interactions with ants but interactions with the blank mimics will not. Statistical analysis was conducted using R version 2.12.1.

## Results

Mimics were treated with similar amounts of hydrocarbon and oleic acid. Forager mimics were treated with a mean of 0.153+/−0.180 (standard deviation (SD)) cuticular hydrocarbon per mimic. Seed mimics contained a mean of 2.57+/−3.39 (SD) micrograms of oleic acid per mimic. Mimics treated with both forager and seed odor contained a mean of 0.296+/−0.151 (SD) micrograms of hydrocarbon per mimic and 2.233+/−2.713 (SD) microgram of oleic acid per mimic.

Quantitative analysis of mimics also showed that we treated mimics with biologically-relevant amounts of hydrocarbon and oleic acid. An ant-equivalent of cuticular hydrocarbon for *P. barbatus* is 9 micorgram [20]. Thus, mimics were coated with about 0.03 of one-ant equivalent of hydrocarbon. This amount of hydrocarbon is within the detection limits of ant antennae and would not be considered a “pharmacological” dose of hydrocarbon considering the small size of mimics relative to ants [26]. Mimics weighed on average 0.00629 g. Thus, oleic acid treated mimics contained on average 0.355 mg of oleic acid/g of mimic which falls within a range of oleic acid present in real seeds (range: 0.20 mg of oleic acid/g of seed to 16.00 mg oleic acid/g of seed) [22].

**Figure 2:**
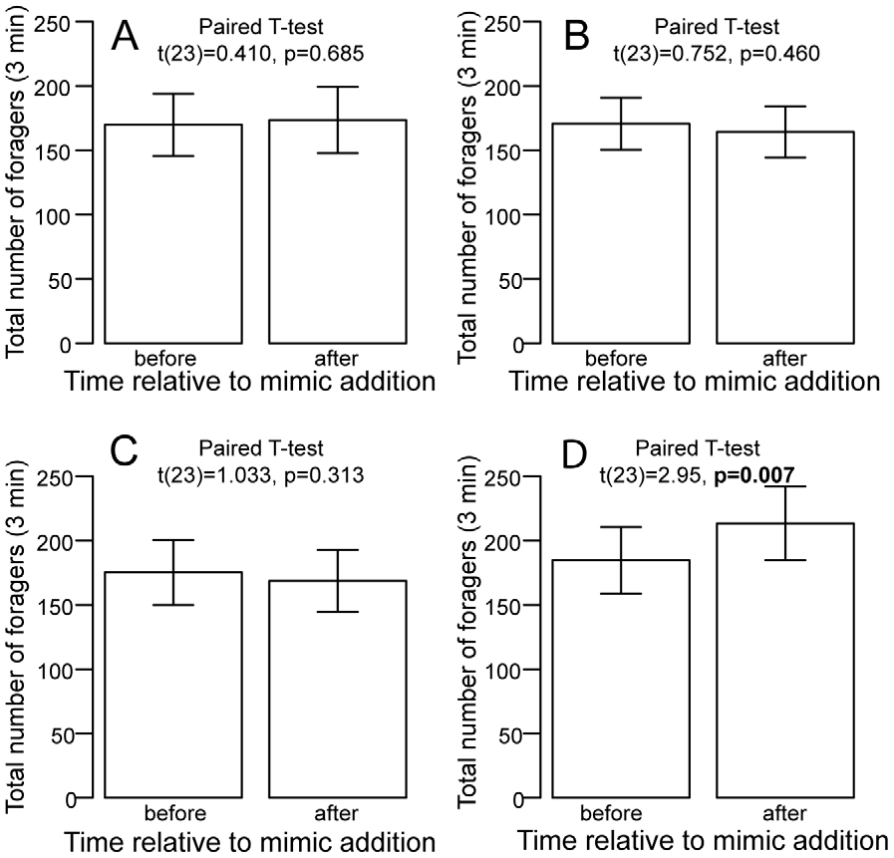
Mean number of foragers per unit time (+/− standard error of mean) before and after addition of seed mimics to nest entrances. A) Addition of blank seed mimics. B) Addition of forager cuticular hydrocarbon seed mimics. C) Addition of oleic acid seed mimics. D) Addition of seed mimics treated with both forager cuticular hydrocarbons and oleic acid. * indicate statistical significant differences among means (p<0.0125).

The rate of outgoing foragers increased in response to mimics treated with the combination of seed odor (oleic acid) and forager odor (cuticular hydrocarbons) (paired-t test t(23) = 2.965, p = 0.007 (2-sided); Fig. 2D). This result is statistically significant when using the adjusted α = 0.0125 after Bonferroni correction. We did not detect a significant change in the rate of outgoing foragers in response to the addition of blank mimics (t (23) = 0.410 p = 0.685 (2-sided); Fig. 2A); in response to the addition of mimics treated with oleic acid alone (t (23) = 0.752, p = 0.460 (2-sided); Fig. 2B); and in response to the addition of forager cuticular hydrocarbon mimics alone (t (23) = 1.033, p = 0.313 (2-sided); Fig. 2C).

We found a significant difference among treatments in the mean change in foraging rate before and after the addition of mimics (repeated-measures ANOVA, F_3,20_ = 4.283, p<0.017). Addition of mimics with a combination of forager and seed odors led to a mean increase of 28.7 (+/−9.7 SEM) foragers per 3 min, while the mean change in foraging rate was only 3.7 (+/−9.0) in response to blank mimics, −6.3 (+/−8.4) in response to seed odor mimics, and −6.5 (+/−6.3) in response to forager odor mimics.

**Figure 3:**
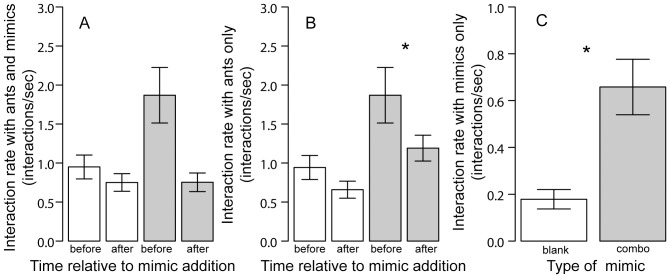
Mean interaction rate (interactions/second; +/− standard error of mean) in the nest entrance with A. ants and mimics before and after addition of blank mimics (white) or combination of forager and seed odor mimics (gray); B. only with ants before and after addition of blank mimics (white) or combination of forager and seed odor mimics (gray); C. only with mimics in blank (white) or combination of forager and seed odor (gray) treatments. * indicates p-value<0.05.

Interactions with mimics bearing the odors of foragers and seeds appeared to have the same effect as interactions with ants. Interaction rate with both ants and mimics did not change after adding either blank mimics or mimics treated with a combination of forager and seed odor (Table 1, Figure 3A). Interaction rate with ants only did not change in response to adding blank mimics but significantly decreased when adding combination of forager and seed odor mimics (Table 1, Figure 3B).

**Table 1:** Results of the ANOVAs comparing the interaction rate with ants and mimics or with ants only before and after the addition of mimics, see also figure 3 A,B.

		overall model statistics			period (before/after mimic addition)			Colony^1^		
		adjusted R2	F	P	DF	T	P	DF	T	P
interactions with both ants and mimics	blank mimics	0.04	1.74	0.19	1	1.29	0.2	1	1.3	0.2
	combo mimics	0.31	9.7	**<0.001**	1	0.4	0.66	1	4.38	**<0.001**
interactions with ants only	blank mimics	0.09	2.86	0.07	1	1.86	0.07	1	1.46	0.15
	combo mimics	0.4	14.12	**<0.0001**	1	2.18	**0.03**	1	4.85	**<0.0001**

1 Colony differences result from differences in overall interaction rate and not from differences in how they responded to the mimics. Trends of the response were the same in direction and significance for both colonies.

Foragers interacted more with mimics coated with the combined odor of foragers and oleic acid than with blank mimics (Figure 3C; ANOVA: overall model: adjusted R^2^ = 0.24, F = 5.14, p = 0.014, treatments (blanks/combo): DF = 1, T = 3.03, p = 0.006; colony: DF = 1, T = 0.35, p = 0.7).

## Discussion

Foragers returning with seeds stimulate the foraging activity of red harvester ant colonies because inactive foragers respond to their interactions with the combined odors of foragers and seeds. Our results indicate that outgoing foragers, waiting inside the nest to leave on the next trip, distinguish a returning forager holding food from one without food using chemical cues. Food odor cues alone are not sufficient to stimulate foraging activity; the rate of outgoing foraging did not increase in response to mimics treated with the odor of food. Forager cues alone were also not sufficient to stimulate foraging activity; the rate of outgoing foraging did not increase in response to mimics treated with the odor of foragers.

We used oleic acid to mimic the odor of seeds because it is found ubiquitously in plants, including seeds and elaiosomes, and it is one of the most abundant fatty acids found in seeds [22]; [27]–[29]. Oleic acid is also found in fatty-acid mixtures used by other ant species to mediate seed collection and distribution [30]–[31]. In harvester ants, oleic acid has been implicated as a releaser of the removal of corpses from the nest mound, often referred to as the necrotic response [32]. However, Gordon [21] showed that the response of harvester ant workers (*P. badius*) to oleic acid treated mimics varied among task groups; foragers treated the mimics as food while nest maintenance workers took them to the refuse pile. Oleic acid has never been observed to elicit a nestmate recognition response, because polar lipids found on the cuticle of *P. barbatus*, including fatty acids, do not provide any relevant nestmate recognition cues [33]–[34].

Interactions between foragers inside the nest link the rate at which outgoing foragers leave the nest to the rate of return of foragers with food. Previous work shows that when returning foragers were deprived of their food and allowed to return to the nest without it, foraging activity decreased [13]. When foragers returning without food were prevented from arriving at the nest, there was no effect on foraging activity [7], while a decrease in the numbers of foragers returning with food decreases foraging activity [7]; [24]; [35]. Here we found that ants inside the nest interacted more with mimics that combined forager and seed odors than with blank controls, and such interactions replaced interactions with foragers.

The result here that the odors of foragers and food are both needed suggests that the crucial encounters between returning foragers and those waiting to leave the nest occur as soon as the returning forager with food enters the nest, before it drops its load. Two other results suggest that inactive foragers respond to very recent encounters with returning foragers. First, foragers require a rapid rate of return of patrollers [16], about 1 per 10 sec, to leave the nest for the first time in the morning, indicating there is a short window during which foragers react to encounters with returning ants. Second, foraging activity responds very quickly, within minutes, in response to a change in forager return rate [23]–[24]; [35]–[36].

By using both the hydrocarbon profile of foragers and of seeds to stimulate foraging, colonies are using the best possible measure of food availability. Foragers drop their seeds when they enter the nest, so a forager with a seed has just returned from its trip outside the nest. The use of the combined cue, the odors of foragers and of seeds, ensures that foraging activity is closely linked to food availability. The return of successful foragers provides the most accurate measure of how long it takes to find food that day. Most of a forager's trip outside the nest is search time [9] so the length of a foraging trip depends on how long the forager has to search for food; the more food available, the shorter the trip.

The rate at which inactive foragers encounter seeds alone would not provide a very precise measure of food availability. Field observations with a videoscope show that during the foraging period, seeds are scattered around inside the entrance tunnel, as other ants, possibly not foragers, move the seeds down into the nest for storage (Gordon, unpublished data). Similarly, in the laboratory ants from deeper inside the nest come to the entrance chamber, collect the seeds, and carry them back inside to the storage chambers. How many seeds an inactive forager encounters depends not only on how quickly food is coming in, but also on how quickly seeds are moved out of the entrance tunnel and chamber. Thus the current rate at which food is coming into the nest is better reflected in the rate at which returning foragers are entering the nest with seeds than in the amount of seeds distributed around the entrance tunnel.

It is remarkable that the response was so robust to mimics treated with the odors of foragers and seeds, even though mimics were dropped at the nest entrance, not deeper in the entrance tunnel where foragers normally interact with returning successful foragers. We relied on other ants to carry the mimics into the tunnel, thus simulating an increase in the return of successful foragers. Thus by adding mimics, we merely enhanced, but did not fully determine, the rate of interaction with returning foragers. Indeed, as we observed directly, interaction rate with both mimics and ants did not significantly change before and after adding the mimics. However, the seed and forager odor mimics, but not the blank mimics, reduced the interaction rate with ants, replacing these with interactions with mimics.

Chemical cues used by ants are often composed of mixtures of multiple chemicals, and recent work shows that combinations of many chemicals can provide information to ants that individual compounds or groups of similar structures cannot [20]. Many ant species use combined chemical cues, including those produced by ants and by food, in foraging behavior. For example, foragers of *Cataglyphis fortis*, a desert ant that forages individually for dead arthropods and, like the red harvester ant, does not use pheromone-recruitment trails to mass recruit to prey, uses mixtures of volatile odor cues associated with different ground structures to locate the nest [37]. Workers of the carpenter ant, *Camponotus pennsylvanicus*, use pheromone trails to recruit foragers to food sources, and also learn airborne volatile cues from plants to locate food resources [38]. Foragers of the leaf cutting ant, *Acromyrmex lundi*, recruit other foragers to a food source using chemical cues specific to that food source [39].

The use of a combination of simple cues makes it possible for red harvester ant colonies to make an accurate and rapid adjustment of foraging activity that corresponds to the current availability of food. By using the combined odors of forager cuticular hydrocarbons and of seeds, the colony captures precise information, on the timescale of seconds about the current availability of food.

## References

[1] Gordon DM (2007) Control without hierarchy. Nature 4468: 143. 10.1038/446143a 17344838

[2] O'Donnell S (2001) Worker biting interactions and task performance in a swarm-founding eusocial wasp (*Polybia occidentalis*, Hymenoptera: Vespidae). Behav Ecol 12: 353–359.

[3] Biesmeijer JC, van Nieuwstadt MGL, Lukacs S, Sommeijer MJ (1998) The role of internal and external information in foraging decisions of *Melipona* workers (Hymenoptera: Meliponinae). Behav Ecol Sociobiol 42: 107–116.

[4] Fernandez PC, Gil M, Farina WM (2003) Reward rate and forager activation in honeybees: recruiting mechanisms and temporal distribution of arrivals. Behav Ecol Sociobiol 54: 80–87.

[5] Camazine S (1993) The regulation of pollen foraging by honey-bees – How foragers assess the colony need for pollen? Behav Eco Sociobiol 32: 265–272.

[6] Grüter C, Balbuena MS, Farina WM (2008) Informational conflicts created by the waggle dance. Proc R Soc Lond B Biol Sci 275: 1321–2327. 10.1098/rspb.2008.0186 PMC2602683 18331980

[7] Schafer RJ, Holmes S, Gordon DM (2006) Forager activation and food availability in harvester ants. Anim Behav 71: 815–822. 10.1016/j.anbehav.2013.05.012 PMC3767282 24031094

[8] Gordon DM, Kulig AW (1996) Founding, foraging and fighting: colony size and the spatial distribution of harvester ant nests. Ecology 77: 2393–2409.

[9] Beverly B, McLendon H, Nacu S, Holmes S, Gordon DM (2009) How site fidelity leads to individual differences in the foraging activity of harvester ants. Behav Ecol 20: 633–638.

[10] Gordon DM (1995) The development of an ant colony's foraging range. Anim Behav 49: 649–659.

[11] Gordon DM (1993) The spatial scale of seed collection by harvester ants. Oecologia 95: 479–487. 28313287 10.1007/BF00317431

[12] Greene MJ, Gordon DM (2007) How patrollers set foraging direction in harvester ants. Am Nat 170: 943–948. 18171176 10.1086/522843

[13] Gordon DM (1991) Behavioral flexibility and the foraging ecology of seed-eating ants. Am Nat 138: 379–411.

[14] Pinter-Wollman N, Wollman R, Guetz A, Holmes S, Gordon DM (2011) The effect of individual variation on the structure and function of interaction networks in harvester ants. J R Soc Interface 8: 1562–1573 DOI 10.1098/rsif.2011.0059. 21490001 10.1098/rsif.2011.0059 PMC3177612

[15] Greene MJ, Gordon DM (2003) Cuticular hydrocarbons inform task decisions. Nature 423: 32. 12721617 10.1038/423032a

[16] Greene MJ, Gordon DM (2007) Interaction rate informs harvester ant task decisions. Behav Ecol 18: 451–455.

[17] Martin SJ, Zhong W, Drijfhout FP (2009) Long-term stability of hornet cuticular hydrocarbons facilitates chemotaxonomy using museum specimens. Biol J Linn Soc 96: 732–737.

[18] Wagner D, Brown MJF, Broun P, Cuevas W, Moses LE, Chao DL, Gordon DM (1998) Task-related differences in the cuticular hydrocarbon composition of harvester ants, *Pogonomyrmex barbatus* . J Chemical Ecol 24: 2021–2037.

[19] Nelson DR, Blomquist GJ (1995) Insect Waxes. In: Hamilton, R J., editors. Waxes: Chemistry, Molecular Biology and Functions. Bridgewater, England: The Oily Press. pp. 1–90.

[20] Greene MJ, Gordon DM (2007) Structural complexity of chemical recognition cues affects the perception of group membership in the ants *Linepithema humile* and *Aphaenogaster cockerelli* . J Exp Biol 201: 897–905. 10.1242/jeb.02706 17297148

[21] Gordon DM (1983) Dependence of necrophoric response to oleic acid on social context in the harvester ant, *Pogonomyrmex badius* . J Chem Ecol 9: 105–111. 24408623 10.1007/BF00987774

[22] Kanchanamayoon W, Wipada K (2007) Determination of Some Fatty Acids in Local Plant Seeds. Chiang Mai J Sci 34: 249–252.

[23] Gordon DM, Holmes S, Nacu S (2008) The short-term regulation of foraging in harvester ants. Behav Ecol 19: 217–222. 10.1093/beheco/arq218 PMC3071749 22479133

[24] Gordon DM, Guetz A, Greene MJ, Holmes S (2011) Colony variation in the collective regulation of foraging by harvester ants. Behav Ecol 22: 429–435. 22479133 10.1093/beheco/arq218 PMC3071749

[25] Adler FR, Gordon DM (2003) Optimization, conflict, and non-overlapping foraging ranges in ants. Am Nat 162: 529–543. 14618533 10.1086/378856

[26] Ichinose K, Lenoir A (2005) Detecting nestmate recognition patterns in the fission-performing ant *Aphaenogaster senilis*: A comparison of different indices. Insect Soc 57: 453–455.

[27] Copeland LO and McDonald MB (2001) Chemistry of Seeds. In: Copeland, LO and McDonald, MB, editors. Principles of Seed Science and Technology. Norwall, Massachusetts: Kluwer. pp. 48–49.

[28] Laffargue A, de Kochko A, Dussert S (2007) Development of solid-phase extraction and methylation procedures to analyze free fatty acids in lipid-rich seeds. Plant Physiol Biochem 45: 250–257. 17360190 10.1016/j.plaphy.2007.01.012

[29] Vioque J, Pastor JE, Vioque E (2003) Fatty acid composition of seed oil triglycerides in *Coincya* (Brassicaceae). J Chem Ecol 70: 1157–1158.

[30] Boulay R, Coll-Toledano J, Manzaneda J, Cerdá X (2006) Geographic variations in *Helleborus foetidus* elaiosome lipid composition: implications for dispersal by ants. Chemoecol 16: 1–7.

[31] Boulay R, Coll-Toledano J, Manzaneda J, Cerdá X (2007) Geographic variations in seed dispersal by ants: are plant and seed traits decisive? Naturwissenschaften 94: 242–246. 17119907 10.1007/s00114-006-0185-z

[32] Wilson EO, Durlach NI, Roth LM (1958) Chemical releasers of necrophoric behavior in ants. Psyche 65: 108–114.

[33] Nelson DR, Tissot M, Nelson LJ, Flatland CL, Gordon DM (2001) Novel wax esters and hydrocarbons in the cuticular surface lipids of the red harvester ant, *Pogonomyrmex barbatus* . Comp Biochem Mol Biol 128: 575–595. 10.1016/s1096-4959(00)00354-7 11250553

[34] Wagner D, Tissot M, Cuevas W, Gordon DM (2000) Harvester ants utilize cuticular hydrocarbons in nestmate recognition. J Chem Ecol 26: 2245–2257.

[35] Gordon DM (2002) The regulation of foraging activity in harvester ant colonies. Am Nat 159: 509–518. 18707433 10.1086/339461

[36] Sturgis SJ, Greene MJ, Gordon DM (2011) Hydrocarbons on harvester ant (*Pogonomyrmex barbatus*) middens guide foragers to the nest. J Chem Ecol 37: 512–524. 10.1007/s10886-011-9947-y 21494855

[37] Steck K, Hansson B, Knaden M (2009) Smells like home: Desert ants, *Cataglyphis fortis*, use olfactory landmarks to pinpoint the nest. Front Zool 6: 5. 19250516 10.1186/1742-9994-6-5 PMC2651142

[38] Helmy O, Jander R (2003) Topochemical learning in black carpenter ants (*Camponotus pennsylvanicus*). Insectes Soc 50: 32–37.

[39] Bollazzi M, Roces F (2011) Information Needs at the Beginning of Foraging: Grass-Cutting Ants Trade Off Load Size for a Faster Return to the Nest. PLoS ONE 6 (3) e17667 doi:10.1371/journal.pone.0017667. 21408014 10.1371/journal.pone.0017667 PMC3052370

